# Topical Agents in Biofilm Disaggregation: A Systematic Review and Meta-Analysis

**DOI:** 10.3390/jcm13082179

**Published:** 2024-04-10

**Authors:** Alessia Pardo, Vera Fiorini, Alessandro Zangani, Paolo Faccioni, Annarita Signoriello, Massimo Albanese, Giorgio Lombardo

**Affiliations:** Section of Oral and Maxillofacial Surgery, Department of Surgical Sciences, Dentistry, Gynecology and Pediatrics, University of Verona, 37124 Verona, Italy; alessia.pardo@univr.it (A.P.); veraunivr@gmail.com (V.F.); paolo.faccioni@univr.it (P.F.); annarita.signoriello@univr.it (A.S.); massimo.albanese@univr.it (M.A.); giorgio.lombardo@univr.it (G.L.)

**Keywords:** periodontitis, periodontal debridement, adjunctive therapy, desiccant agent, desiccation, sodium hypochlorite

## Abstract

**Background**: to evaluate the effectiveness of different topical agents in biofilm disaggregation during non-surgical periodontal therapy. **Methods**: the search strategy was conducted according to the PRISMA 2020 on Pubmed, Cochrane Library, Scopus, and Web of Science, and it was registered in PROSPERO, ID: CRD42023474232. It included studies comparing non-surgical periodontal therapy (NSPT) with and without the application of topical agents for biofilm disruption. A risk of bias analysis, a qualitative analysis, and a quantitative analysis were performed. **Results**: out of 1583 records, 11 articles were included: 10 randomized clinical trials and one retrospective analysis. The total number of participants considered in the 11 articles included in the study was 386. The primary outcomes were probing pocket depth (PPD), clinical attachment level (CAL), and bleeding indices. The secondary outcomes were plaque indices, gingival recessions, and microbiological parameters. The meta-analysis revealed the following: [Weighted mean difference (WMD): −0.37; 95% confidence interval (CI) (−0.62, −0.12), heterogeneity I^2^: 79%, statistical significance *p* = 0.004]. **Conclusions**: the meta-analysis of probing pocket depth reduction (PPD) between baseline and follow-up at 3–6 months showed a statistically significant result in favor of sulfonated phenolics gel. The scientific evidence is still limited and heterogeneous; further randomized clinical trials are required.

## 1. Introduction

According to the American Academy of Periodontology, periodontitis is a chronic disease with a multifactorial etiology. Periodontitis is associated with the presence of dysbiotic biofilm and characterized by a progressive loss of attachment, bone resorption, and formation of a periodontal pocket [1]. Periodontal disease thus leads to the destruction of both supporting soft and hard tissues and the possible loss of dental elements over time. Furthermore, recent studies found a correlation between oral biofilm and systemic inflammation, e.g., cardiovascular diseases [2]. 

Non-surgical periodontal therapy is currently the gold standard in periodontal treatment to reduce periodontal pathogens in order to achieve a reduction in the probing pocket depth, to eliminate inflammation, and to stop the disease progression [3]. Non-surgical periodontal therapy is carried out with manual instruments or mechanical ultrasonic or sonic instruments [4].

However, in specific clinical conditions, mechanical biofilm removal alone is not enough: in these cases, periodontal debridement does not provide sufficient achievement of satisfying clinical outcomes [5,6]. These cases may include, for example, deep pockets, the involvement of furcation, and anatomical areas that are difficult to access [7,8]. There is evidence in the scientific literature about the introduction of additional therapies aiming to improve the objectives that can be achieved with non-surgical mechanical periodontal therapy alone [9]. Examples of frequently used topical applications include antiseptic substances such as chlorhexidine (one of the most widely used oral antimicrobial agents, available in different formulations), antibiotics in gel, fibers, or other formulations. 

Because periodontitis a biofilm-mediated disease, antibiotic treatment in periodontal patients is typically selected empirically or using technical methods. These approaches are directed towards establishing the levels of different periodontal pathogens in periodontal pockets to determine the appropriate antibiotic treatment. However, current methods are costly and do not consider the antibiotic susceptibility of the entire subgingival biofilm [10]. 

Furthermore, controversies associated with local delivery are also reported: induction of bacterial resistant strains, the efficacy of systemic versus local drug delivery, and whether local drug delivery should function as an alternative or as an adjunct to conventional treatment [11].

Moreover, the additional use of ozone therapy, laser, and photodynamic therapy has been reported [12,13,14]. Because of their action against anaerobic bacteria, the use of these physical agents is currently being discussed in cases of post-extraction complications and in patients with chronic gingivitis, periodontitis, and periodontal abscesses [15]. Finally, topical disaggregating agents have been applied into the periodontal pockets before the mechanical instrumentation. Because their primary target is the bacterial matrix, their purpose is to disaggregate this matrix. Consequently, it is then easier to remove biofilm deposits with mechanical debridement [13,16].

In light of these considerations, the aim of this systematic review was to analyze and compare different topical active ingredients as additional therapy during non-surgical treatment of periodontal disease, with specific attention to their biofilm disaggregation activity. 

## 2. Materials and Methods

This systematic and meta-analysis review was conducted in accordance with the criteria presented in the latest version of the Preferred Reporting Items for Systematic reviews and Meta-Analyses (PRISMA 2020) [17]. We also report that this review is registered under number CRD42023474232 with PROSPERO, the international prospective register of systematic reviews.

### 2.1. Focused Question

The current review attempts to answer the following question: in patients with periodontal disease (referred to as the patient), is there sufficient and adequate evidence in the scientific literature that these topical agents (referred to as the intervention) lead to an improvement in clinical and microbiological parameters (referred to as the outcome) compared to standard periodontal therapy (referred to as the control)?

### 2.2. Search Strategy

An electronic search was implemented to retrieve all relevant studies. 

The search was carried out using the following database: PubMed, Cochrane Library, Scopus, and Web of Science.

Publications published from January 2008 to October 2023 were considered, including randomized clinical trials (RCTs) and retrospective analyses. Relevant keywords and Boolean operators (AND, OR, NOT) were used to implement the following search string: (periodontitis OR periodontal disease) AND (chemical cleansing OR adjunctive therapy OR topical treatment OR subgingival irrigation OR topical agent OR desiccant agent OR chloramine OR sodium hypochlorite OR Perisolv OR hypochlorite) NOT (antibiotic OR systemic disease OR laser OR photodynamic OR orthodontic OR mouthwash OR rinse OR systematic review OR alendronate OR simvastatin OR atorvastatin OR rosuvastatin OR toothpaste) AND (randomized controlled study OR clinical trial OR retrospective analysis).

### 2.3. Screening and Selection

The search was carried out by two reviewers (VF e AP) who worked independently through the screening of titles, abstracts, and full text of the studies obtained from the searches by applying the inclusion and exclusion criteria that were already established. Any disagreement concerning eligibility was resolved by discussion between the parties. 

The studies included in the search strategy were as follows: randomized controlled clinical trials (RCTs), prospective studies, and retrospective studies. In vitro studies and studies on animals were excluded. The studies that fulfilled all the inclusion criteria were processed for data extraction.

The inclusion criteria were:

- Health status ASA I;
- Patients with periodontal disease: periodontitis stage I and II;
- Topical agents with an action of biofilm disaggregation;
- Primary outcomes: clinical outcomes of PPD (pocket probing depth), CAL (clinical attachment level), BOP (bleeding on probing);
- Secondary outcomes: clinical outcomes of PI (plaque index) and REC (recession) and microbiological outcomes.

The exclusion criteria were:

- Patients treated with surgical therapy for periodontitis;
- Dental implants;
- Systemic diseases (ASA II, III);
- No previous antibiotic prophylaxis;
- Topical agents with a chemical action on biofilm (systemic administration of drugs, antibiotics, probiotics);

Topical agents with a physical action on biofilm (laser, ozone therapy).

### 2.4. Risk of Bias Assessment

Two reviewers (VF and AP) performed the risk of bias analysis of the included studies using two different tools.

The first one was the RoB 2 tool [18] for RCTs, and the second one was the ROBINS-I tool for retrospective analysis [19]. The scoring was based on different domains and could be scored as unclear, low risk of bias, or high risk of bias. The following items were evaluated: random sequence generation, allocation concealment, blinding of participants and personnel, blinding of outcome assessment, incomplete outcome data, selective reporting, and others. It was “a priori” decided that the domains 5.2 and 5.3 were not answered because the risk of bias assessment was made at study level and not for every outcome. A study was estimated to be at a high risk of bias if at least one domain had a high risk of bias, at an unclear risk of bias if at least one domain was unclear and none were high, and at a low risk of bias if all domains were assessed as being at low risk of bias. 

### 2.5. Data Extraction

The reviewer extracted the details on the characteristics of the studies using an Excel paper. 

The following data were collected: author, year of publication, study design, country, sample number, mean age, gender, test and control interventions, follow-up, outcomes.

The means and standard deviations were extracted if available.

Methods outlined by *The Cochrane Handbook* were used for imputing missing standard deviation. Imputed standard deviations were calculated for studies that provided a mean and confidence interval. If the sample size was small (<60), then the confidence intervals were calculated using a value from a t distribution, obtained from the tables of the t distribution, with degrees of freedom that were equal to the group sample size minus 1 (n − 1). If the standard deviation (SD) could not be calculated, data were imputed from a similar study included in this review through the correlation index.

**Figure 1:**
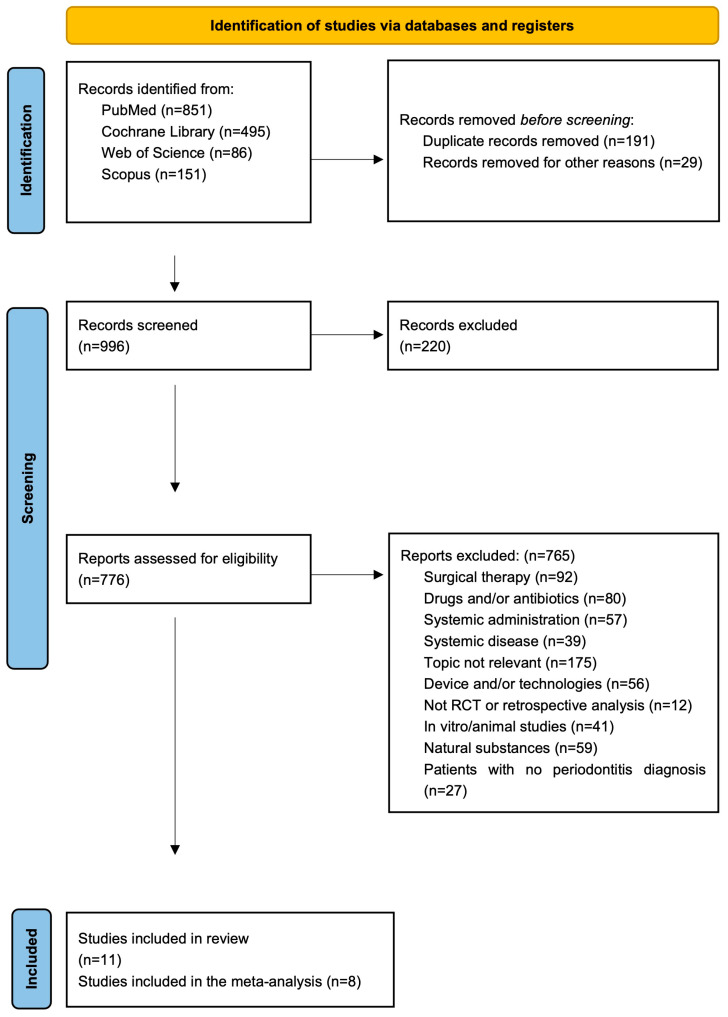
PRISMA flow diagram of the search and selection process [17].

All the formulae used are represented in Figure 1.

### 2.6. Data Analysis

The meta-analysis was performed using Review Manager Web [20]. 

The meta-analysis resulted in a 95% confidence interval (CI) using the inverse variance method and a random effect model. A meta-analysis was performed on subgroups. The heterogeneity was interpreted following *The Cochrane Handbook* guidelines: 0–40% indicated that heterogeneity was negligible, 30–60% indicate that heterogeneity was moderate, 50–90% indicated that heterogeneity was substantial, and 75–100% indicated that heterogeneity was considerable [21].

### 2.7. Grading the Body of Evidence

The grade was obtained with GRADEpro GDT [22]. Two reviewers (VF and AP) rated the certainty of the evidence according to the following aspects: risk of bias, inconsistency, indirectness, imprecision, publication bias, large effect, plausible confounding, and dose–response gradient. Any disagreement between the two reviewers was resolved after additional discussion.

## 3. Results

### 3.1. Search and Selection Results

The search conducted on Pubmed, Cochrane Library, Scopus, and Web of Science resulted in 1583 papers. Filters concerning year of publication (2008–2024) and type of publication (article) were applied and resulted in 996 papers. After the removal of duplicates (191) and inaccessible papers (29), a total of 776 studies were screened.

The screening of the titles, abstracts, or full text resulted, for both reviewers, in the inclusion of 11 papers. The studies were all RCTs; only one was a retrospective analysis. Figure 1 shows the PRISMA flow diagram. 

### 3.2. Characteristics of Included Studies

The studies included in this systematic review were conducted in eight different countries: Italy, Romania, Switzerland, Syria, India, Netherlands, Lithuania, and Germany.

They were published between 2015 and 2024.

Ten out of eleven were randomized controlled studies, and one was a retrospective analysis of case series.

The RCTs were comparative studies performed in periodontal patients to evaluate the adjunctive use of topical agents in biofilm disaggregation during non-surgical periodontal therapy and standard therapy. 

In all the studies, the number of participants ranged from 16 to 56, with an age average of 48.27 years and a proportion of male and female of 47.43% and 52.57%, respectively. 

All studies assessed parameters at baseline and at different follow-up times: eight studies performed a follow-up at 6 months, six at 3 months, four at 12 months. 

Seven studies included the application of sodium hypochlorite [23,24,25,26,27,28,29], and four studies used a desiccant agent with sulfonated phenolics [30,31,32,33]. The retrospective study describes the application of sodium hypochlorite [25].

All the 11 studies’ characteristics are summarized in Table 1.

### 3.3. Sodium Hypochlorite

Sodium hypochlorite was applied into the periodontal pockets as a liquid solution only in one study.

One author used a gel of 0.05% NaOCl, whereas the other five studies applied a new gel composed by NaOCl plus amino acids. In this way, it was able to create chloramine which penetrates the biofilm. Moreover, the high pH (pH = 12) of the gel enhances the disaggregation effect of the NaOCl.

Furthermore, to accelerate the healing of soft tissues, two studies used hyaluronic acid after the application of NaOCl gel (Clean & Seal) into the periodontal pockets.

The NaOCl gel was applied before the traditional non-surgical therapy in four studies, while in two studies it was applied after the therapy (see Table 2).

### 3.4. Desiccant Agent

The desiccant agent is a gel with strong hygroscopic properties, and in consequence, it absorbs the water from the organic matrix, leading to the denaturation of the biofilm structure. This sulfonated gel was applied before the traditional non-surgical therapy in all the included studies; in this way, it became easier to remove the deposits mechanically. The gel was kept into the periodontal pockets for a range of time between 20 s and 60 s (see Table 2).

### 3.5. Risk of Bias Assessment

Analysis of the risk of bias was carried out for all the included studies.

The retrospective analysis showed an overall low risk of bias.

Five out of ten RCTs showed a low risk of bias, while the other five demonstrated an unclear risk of bias.

The domains with some concern risks were the random sequence generation and allocation concealing and the registration of the protocol and its concordance with the published article.

**Figure 2:**
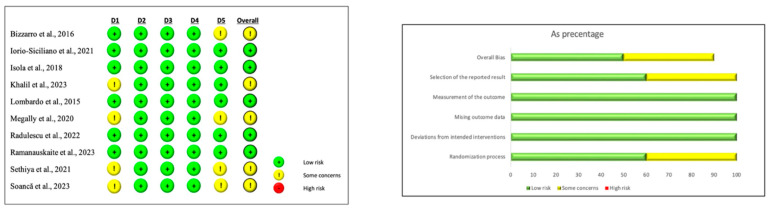
RoB 2 tool (risk of bias assessment) for RCTs [23,24,26,27,28,29,30,31,32,33].

Figure 2 shows the risk of bias assessment results.

### 3.6. Study Outcomes Results: Qualitative Analysis

All the studies performed the analysis of PPD, CAL and BOP, with nine of them asserting an improvement in PPD and CAL from baseline to follow-up for both the treatment groups. Five studies out of nine found a significative difference favoring the test group in at least one of the two indices (PPD and/or CAL).

Regarding BOP, it was found to be improved in all the studies included; seven studies out of eleven found a significative difference favoring the test intervention, while one study asserted that the control intervention provided a better reduction at 12 months.

The PI was analyzed in eight studies, and all of them demonstrated a statistically significant reduction in both treatments from baseline to follow-up. Only two studies showed a significant difference between the interventions, both favoring the test group.

The REC was the last index analyzed: just one study found a statistically significant difference between the studies; this difference favored the topical agent.

Five out of eleven studies included [23,26,28,30,31] analyzed microbiological changes over time in the test and control groups, respectively. Studies by Radulescu [28] and Megally [23] did not show any statistically significant differences between the groups at any follow-up time. In contrast, the other studies showed a statistically significant difference between the groups.

### 3.7. Meta-Analysis

Eight studies were included for quantitative analysis, and meta-analysis was performed in two subgroups to evaluate the outcome of the two different topical agents on the reduction in probing pocket depth (ΔPPD) from baseline to the follow-up at three or six months. Table 3 shows the means and SDs of the PPD change from baseline to follow-up.

**Figure 3:**
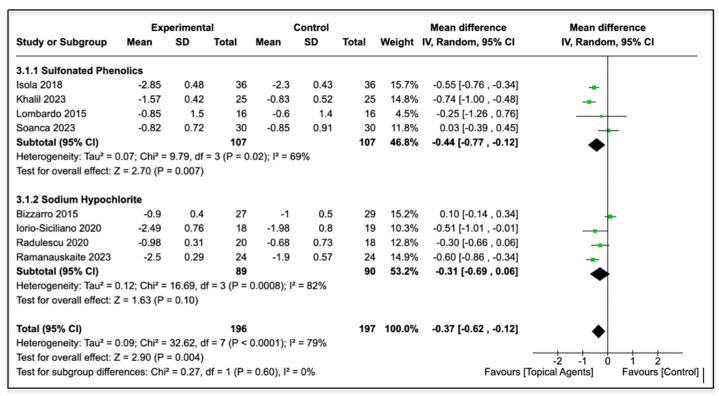
Forest plot of the meta-analysis [24,26,28,29,30,31,32,33].

Figure 3 provides a summary of the meta-analysis outcomes. In almost all instances, the baseline scores were not statistically different. The plaque scores were higher in the test group in one study [29]. Moreover, the BOP was higher in the test group in one study [24], while other two studies reported a higher value in the control group [28,31].

The total result shows the following values: [WMD: −0.37; 95% IC (−0.62, −0.12) with heterogeneity I^2^: 79% and statistical significance *p* = 0.004].

The first subgroup analysis performed showed sulfonated phenolics and sulfuric acid gel, and it highlighted a [WMD: −0.44; 95% IC (−0.77, −0.12) with heterogeneity I2: 69% and statistical significance *p* = 0.007. The second subgroup analysis performed showed sodium hypochlorite, and it showed a [WMD: −0.31; 95% IC (−0.69, −0.06) with heterogeneity I2: 82% and statistical significance *p* = 0.10].

### 3.8. Grading the Body of Evidence

The evidence and the strength of the recommendations were evaluated according to GRADEpro GDT.

The estimated risk of bias varied from low to unclear, so reporting bias was considered not serious. Comparisons between the two topical agents indicate moderate certainty evidence for their additional effect over non-surgical periodontal therapy alone. The application of the two topical agents can be recommended to improve PPD reduction over time.

## 4. Discussion

Non-surgical periodontal treatment aims to remove biofilm by manual, ultrasonic, or sonic instruments [4]. However, under specific clinical conditions, mechanical removal alone is not sufficient [5,6], and the permanence of periodontopathogens can lead to residual periodontal pockets [34]. In this paper, it has been shown that different additional therapies can reduce the need for surgical therapy, improving PPD and CAL outcomes [35]. Common antiseptic solutions are not able to directly disaggregate the biofilm, although they inhibit the formation of new bacterial plaque [36]. For this reason, recent developments in periodontal strategies for non-surgical therapy focus not only on the use of antiseptics and antibiotics with bacteria as a target but also on substances able to disaggregate the biofilm [37,38,39,40,41].

From this assumption, the present systematic review has considered alternative topical agents not based on chemical or physical functions, finally selecting, in the search strategy, sodium hypochlorite (NaOCl) and a sulphonated gel. A recent systematic review [37] which considers the same articles included in this paper as regards the application of NaOCl [23,24,26,28], declared that this gel could significantly improve PPD values at 6-month follow-up, while no significant difference was detected after 3 months. Similarly, this systematic review conducted a meta-analysis regarding the PPD parameter as a difference (Δ) between baseline and follow-up, finding a statistically significant difference in the sulphonate gel. On the other hand, the forest plot of NaOCl obtained results lacking statistical significance, even in favor of the test group. More precisely, Bizzarro et al. [26] reported disagreement with three other studies: this difference could be attributed to the fact that NaOCl was used just as a liquid solution instead of being used as a gel with NaOCl and amino acids.

In addition, it is relevant to report that two studies [25,29] implemented a new method, called “Clean & Seal”, which involves the addition of hyaluronic acid, demonstrating its effectiveness in improving clinical parameters. This claim is supported by other evidence in the scientific literature of its restorative and regenerative action, resulting in a lower need for surgical therapy [38,39].

Regarding sulphonated gel, a study by Nardi [40] shows that its use leads to an improvement in inflammatory clinical parameters at one week and at one month of follow-up in cases of periodontal patients suffering from rheumatoid arthritis. Nevertheless, these results are presented in a case report. Zafar [42] assessed elements for extraction and reported that the application of this gel does not appear to significantly improve the removal of calculus in the deep pockets of the posterior elements or elements with complex morphology.

Concerning the potential of sulphonated gel in biofilm removal, Bracke demonstrated, in a case report [43], its effectiveness in plaque disintegration, elimination of pathogenic periodontal bacteria, and prevention of the occurrence of resistant microbial flora. Lauritano [44] considered a sample of 11 patients, in which it was reported that the gel reduced each of the red complex bacteria by 99% after a single application.

Results of the abovementioned studies are in line with the microbiological findings found in this systematic review: Lombardo et al., at the end of the first treatment session, showed a significant reduction (*p* < 0.0001) both for aerobic and anaerobic bacterial species. However, it should be noted that this reduction was recorded for both interventional groups; only at six weeks was it found that a significant reduction occurred in aerobic species in the test group (*p* = 0.02) and that no reduction occurred in the control group [31].

Furthermore, Isola et al. demonstrated a decrease in bacteria of the red complex, in this case, after 15, 30, 60, and 180 days, and such differences remained for up to a year (*p* < 0.001). A recent review [12] showed that non-surgical periodontal treatment associated with chlorhexidine gel did not lead to any statistical significance in the reduction in PPD at 3-month follow-up compared to the control group (0.49 [1.13, 0.14], *p* = 0.05). This is partly in disagreement with the findings of the present review: regarding sulphonated gel, a statistically significant difference was found with respect to the control group (*p* = 0.007), while NaOCl showed no statistical significance (*p* = 0.10).

In terms of BOP, a significant improvement at 3 and 6 months was observed in two studies using Chlorhexidine. One of these showed a bleeding index of 90% and 95% at the baseline, respectively, in the control and test group; at 3 months, the respective values decreased to 21.5% and 5% (with *p* = 0.0001). These findings are consistent with this review, as it can be concluded that a total of six studies [21,25,26,27,28] reported a statistically significant difference in BOP reduction in favor of the test group, even if not at all follow-up intervals. According to Gegout’s review [12], Khalil’s study [32] reported a baseline bleeding index of 70.28% and 71.02% in the control and test group, respectively, decreasing to 44.22% and 22.03% (*p* < 0.001) after 3 months. Iorio-Siciliano [24] also described decreasing bleeding rates at 6 months: in the test group, rates decreased from 85.3% to 2.2%; in the control group, rates decreased from 81.6% to 7.3% (with *p* = 0.001).

Almost all studies demonstrated a significant reduction in PPD from baseline to follow-up, and five studies reported a significant result in favor of the test group; Radulescu and Megally [23,28] showed no statistical significance at any follow-up. Referring to the most recently published reviews about the application of several topical agents’ gels, including antibiotics [12], the meta-analysis failed to demonstrate a significant improvement in PPD at 3 months (0.50 [1.20, 0.20] *p* = 0.16) in sites treated with SRP + metronidazole compared to SRP as monotherapy (placebo gel). In contrast, tetracyclines showed a significant improvement in PPD at 3 months (0.51 [0.71, 0.31], *p* <0.001) compared to control.

Our meta-analysis has shown a statistical significance (−0.37 [−0.62, −0.12], *p* = 0.0004), and therefore, results are similar to evidence found in the literature regarding tetracyclines but not metronidazole. The results of this paper are also consistent with those of a review concerning photodynamic therapy [14], where a difference in PPD was found in favor of the test group compared to the control group. In this study, statistical significance was found in a total of five out of thirteen studies included in a 3-month follow-up. Three studies conducted a further 6-month follow-up appointment, showing again a statistically significant difference in favor of the group treated with photodynamic therapy. Overall, these PPD changes were (−1.79 [−2.26, −1.31] with *p* < 0.00001). Regarding the analysis of microbiological parameters, the same systematic review [14] concludes that the bactericidal efficacy of photodynamic therapy in addition to non-surgical periodontal therapy remains questionable: no statistically significant difference or even any difference between treatment groups could be found. Similarly, in this review, only five studies out of a total of eleven analyzed microbiological changes, with contrasting results.

### Limitations

This systematic review and meta-analysis presents some limitations regarding the evidence included, as a certain degree of heterogeneity emerged from the meta-analysis.

Differences between studies could be attributed to different periodontal case definitions, as well as different stages and grades of periodontal disease considered in each trial. Moreover, a discrepancy in the range of PPD was observed. Two studies considered patients enrolled in supportive periodontal therapy, while the remaining nine did not.

Furthermore, the evaluation of change in BOP or spontaneous bleeding after healing after mechanical or chemical dissolution and removal of soft and hard deposits could be related to tobacco smoking as a possible confounding factor: not all studies have included, among the exclusion criteria, this risk factor closely related to the progression of periodontal disease.

Finally, regarding the application of topical agents, it is relevant to say that in two studies, it was used a liquid solution instead of a viscous formulation.

## 5. Conclusions

Based on the evidence gathered with this systematic and meta-analytical review, it can be concluded that the application of gel with the phenolics sulfonate and sulfuric acid as an adjunctive therapy to non-surgical periodontal therapy was shown to improve clinical and microbiological parameters compared to non-surgical periodontal therapy alone. Therefore, in light of the current scientific evidence and related limitations of the meta-analysis, the qualitative analysis found that the two active ingredients analyzed can be considered as promising topical agents for the disaggregation of biofilm during non-surgical periodontal therapy.

## Figures and Tables

**Table 1:** Characteristics of the included studies.

Authors	Year	Study Design	Country	Sample Number	N Test/Age N Control/Age	Gender	Test and Control Interventions	Follow-Up	Outcome
Bizzarro et al. [26]	2016	RCT, full-mouth	Amsterdam, the Netherlands	56	n 27/47.7 n 29/46.9	F: 20, M: 36	1. BPT + NaOCl 2. BPT + saline solution	12 months	PPD, CAL, BOP, PS
Daniel Diehl et al. [25]	2022	Retrospective analysis	Witten, Germany	29	n 29/54.6	F: 20, M: 9	1. gel NaOCl/aa + UD e/o SRP + HA	6 months	PPD, CAL, BOP, GR
Iorio-Siciliano et al. [24]	2021	RCT, full-mouth	Messina, Italy	37	n 18/53.3 n 19/48.5	F: 21, M: 16	1. MINST + gel NaOCl/aa 2. MINST	6 months	PD, CAL, BOP, FMPS, GR
Isola et al. [30]	2018	RCT, split-mouth	Messina, Italy	36	n 36/46.7	F: 17, M: 19	1. SRP + Hybenx 2. SRP	12 months	PD, CAL, BOP, PS, GR
Khalil et al. [32]	2023	RCT, split-mouth	Damascus, Syria	25	n 25/45.2	F: 15, M: 10	1. SRP + Hybenx 2. SRP	6 months	PPD, RAL, GI, BOP, PLI, GH
Lombardo et al. [31]	2015	RCT, split-mouth	Verona, Italy	16	n 16/55	F: 9, M: 7	1. UD + Hybenx 2. UD	3 months	PPD, CAL, GI, BOP, VPI, GM, microbiology
Megally et al. [23]	2020	RCT, full-mouth	Geneva, Switzerland	32	n 16/61.7 n 16/62.1	F: 11, M 21	1. US + gel NaOCl/aa 2. US	12 months	PD, BOP, GR, microbiology
Radulescu et al. [28]	2022	RCT, full-mouth	Timisoara, Romania	42	n 20/44.60 n 18/50.61	F: 22, M 16	1. UMI + air polish + gel NaOCl/aa 2. UMI + air polish + placebo gel	12 months	PPD, CAL, FMBS, FMPS, GR, microbiology
Ramanauskaite et al. [29]	2023	RCT, full-mouth	Kaunas, Lithuania	48	n 24/47.3 n 24/49.3	F: 35, M 13	1. SRP + NaOCl/aa + HA 2. SRP	6 months	PPD, CAL, BOP, PI
Sethiya et al. [27]	2021	RCT, full-mouth	Maharashtra, India	29	n 11/28.27 n 11/29.09	F: 11, M 11	1. OSFMD + NaOCl gel + mouthwash 2. OSFMD + CHX gel + mouthwash	6 months	PPD, CAL, mSBI, PI
Soancă et al. [33]	2023	RCT, split-mouth	Cluj-Napoca, Romania	36	n 30/44.8	F: 13, M 17	1. SRP + Hybenx 2. SRP	3 months	PD, CAL, GBI, OHI, GR

Abbreviations: RCT: randomized control trial, SRP: scaling and root planing, BPT: basic periodontal therapy, MINST: minimally invasive non-surgical technique, UD: ultrasonic debridement, US: ultrasonic scaling, UMI: ultrasonic mechanical instrumentation, OSFMD: one-stage full-mouth disinfection, NaOCl: Sodium Hypochlorite, HA: Hyaluronic Acid, CHX: Chlorhexidine, PPD: probing pocket depth, BOP: bleeding on probing, CAL: clinical attachment level, RAL: real attachment level, PI: plaque index, PS: plaque score, VPI: visible plaque index, mSBI: modified sulcus bleeding index, GI: gingival index, GM: gingival margin, GR: gingival recession.

**Table 2:** Characteristics of the test and control interventions.

Author	Periodontitis	Study Design	SRP (Test and Control)	Topical Agent (Test)	Topical Agent (Control)
Bizzarro et al., 2016 [26]	CAL ≥ 3 mm PPD ≥ 5 mm	*Full-mouth*	Ultrasound (Hu-Friedy EMS piezon; Hu-Friedy, Chicago, IL, USA); manual instrumentation	After 3 days: NaOCl solution	After 3 days: saline solution
Diehl et al., 2022 [25]	PPD ≥ 5 mm	*Full-mouth*	Ultrasound supra-gingivally; manual instrumentation with Gracey curettes (Deppeler, American Dental Systems, Monaco, Germany)	After SRP: 30–45 s gel NaOCl/aa (Perisolv; Regedent AG, Zurig, Switzerland); further application of the gel as needed After SRP: gel IA (xHyA; hyaDENT BG, Regedent AG, Zurig, Switzerland) Further application within 7 days	No placebo
Iorio-Siciliano et al., 2022 [24]	Stage III/IV, grade A/B PPD ≥ 5 mm	*Full-mouth*	Ultrasound (Instrument PS^®^EMS Electro Medical System S.A., Nyon, Switzerland); Gracey micro-curette (Hu-Friedy^®^, Chicago, IL, USA); polishing	Before SRP: 30 s gel NaOCl/aa (Perisolv^®^, Regedent AG, Zurich, Switzerland) No rinse After SRP: further application of the hyaluronic gel	No placebo
Isola et al., 2018 [30]	Cronic periodontitis, PPD ≥ 5 mm	*Split-mouth*	Ultrasound with insert number 5/6/7 (Satelec Ultrasonics, Acteon, VA, Italy); manual instrumentation (Gracey curettes, ASA Dental, Bozzano, Italy)	Before SRP: 60 s sulfonated gel (HYBENX, oral tissue decontaminant, EPIEN Medical, St Paul, MN, USA); Saline solution removal	Before SRP: 60 s saline solution
Khalil et al., 2023 [32]	Stage III, PPD ≥ 6 mm	*Split-mouth*	Manual instrumentation (CK6 e U-15, ZaffiroTM, Beckum, Germany; Gracey curettes: ZaffiroTM, Germany)	Before SRP: 30 s sulfonated gel (HYBENX, oral tissue decontaminant, EPIEN Medical, St Paul, MN, USA); Saline solution removal	No placebo
Lombardo et al., 2015 [31]	Moderate or severe PPD ≥ 5 mm	*Split-mouth*	Ultrasound (Piezon Master 400, EMS, Nyon, Switzerland) with standards inserts	Before SRP: 45–60 s sulfonated gel (HYBENX, oral tissue decontaminant, EPIEN Medical, St Paul, MN, USA); Saline solution removal	No placebo
Megally et al., 2020 [23]	PPD ≥ 5 mm	*Full-mouth*	Ultrasound (Piezon^®^ LED, EMS Electro Medical System S.A., Nyon, Switzerland)	Before SRP: 30 s gel NaOCl/aa (Perisolv^®^, Regedent AG, Zürich, Switzerland) After SRP: further application of hyaluronic gel	No placebo
Radulescu et al., 2022 [28]	Stage III/IV, PPD ≥ 4 mm	*Full-mouth*	Supragingival ultrasound (EMS Piezon^®^ Master, EMS, Nyon, Switzerland); air polishing (standard air-flow nozzle, AIRFLOW^®^ PLUS powder EMS, Nyon, Switzerland); sites with PPD > 4 mm: ultrasound with subgingival insert (PS, EMS, Nyon, Switzerland)	Before SRP: 30 s gel NaOCl/aa (Perisolv^®^, Regedent AG, Zürich, Switzerland) After 15 min, further application of hyaluronic gel and SRP	Before SRP: placebo
Ramanauskaite et al., 2023 [29]	Stage II-III, grade A/B	*Full-mouth*	Ultrasound (Satelec/Acteon Suprasson Newtron ultrasonic scaler); manual instrumentation (LM SharpDiamond 1/2, 7/8, 11/12, 13/14 SD Gracey curettes and mini-curettes, LM Dental™, Pargas, Finland); polish (Lunos Super Soft, RDA < 5, Dürr Dental, Germany)	Before SRP: 60 s gel NaOCl/aa (Perisolv^®^, Regedent AG, Zurig, Switzerland) Further application of gel as needed (max 2–3 months)After SRP: gel IA (Hyadent^®^ BG, Regedent AG, Zurigo, Switzerland)	No placebo
Sethiya et al., 2021 [27]	PPD ≥ 4 mm and BOP or PPD ≥ 5 mm	*Full-mouth*	SRP in 24 h supra- and subgingivally	After SRP: gel NaOCl 0.05% (5 mL 10% NaOCl and 995 mL sterile water) Gel application for 2 months	After SRP: gel CHX
Soancă et al., 2023 [33]	Stage III/IV, PPD ≥ 4 mm	*Split-mouth*	Ultrasound (Unit-P5 Booster Suprasson-Satelec, Acteon, Mount Laurel, NJ, USA); manual instrumentation: Gracey curettes (Hu-Friedy, Chicago, IL, USA)	Before SRP: 20 s sulfonated gel	No placebo

Abbreviations: PPD, probing pocket depth; s, seconds; NaOCl, sodium hypochlorite; IA, hyaluronic acid; CHX, chlorhexidine.

**Table 3:** Mean and SD of PPD change.

ΔPPD (mm)			
	Control (Mean ± SD)	Test (Mean ± SD)	Follow-Up
Bizzarro et al., 2016 [26]	1 ± 0.5	0.9 ± 0.4	Baseline—6 months
Iorio-Siciliano et al., 2021 [24]	1.98 ± 0.8	2.49 ± 0.76	Baseline—6 months
Isola et al., 2018 [30]	2.3 ± 0.43	2.85 ± 0.48	Baseline—6 months
Khalil et al., 2023 [32]	0.83 ± 0.52	1.57 ± 0.42	Baseline—6 months
Lombardo et al., 2015 [31]	0.6 ± 1.4	0.85 ± 1.5	Baseline—3 months
Radulescu et al., 2022 [28]	0.68 ± 0.73	0.98 ± 0.31	Baseline—3 months
Ramanauskaite et al., 2023 [29]	1.9 ± 0.57	2.5 ± 0.29	Baseline—3 months
Soancă et al., 2023 [33]	0.85 ± 0.91	0.82 ± 0.72	Baseline—3 months

## Data Availability

Data are available from the corresponding authors upon reasonable request.
